# Venomics-Accelerated Cone Snail Venom Peptide Discovery

**DOI:** 10.3390/ijms19030788

**Published:** 2018-03-09

**Authors:** S. W. A. Himaya, Richard J. Lewis

**Affiliations:** IMB Centre for Pain Research, Institute for Molecular Bioscience, The University of Queensland, Queensland 4072, Australia; h.siddhihalu@imb.uq.edu.au

**Keywords:** cone snails, venom, venomics, proteomics, transcriptomics, visualisation

## Abstract

Cone snail venoms are considered a treasure trove of bioactive peptides. Despite over 800 species of cone snails being known, each producing over 1000 venom peptides, only about 150 unique venom peptides are structurally and functionally characterized. To overcome the limitations of the traditional low-throughput bio-discovery approaches, multi-omics systems approaches have been introduced to accelerate venom peptide discovery and characterisation. This “venomic” approach is starting to unravel the full complexity of cone snail venoms and to provide new insights into their biology and evolution. The main challenge for venomics is the effective integration of transcriptomics, proteomics, and pharmacological data and the efficient analysis of big datasets. Novel database search tools and visualisation techniques are now being introduced that facilitate data exploration, with ongoing advances in related omics fields being expected to further enhance venomics studies. Despite these challenges and future opportunities, cone snail venomics has already exponentially expanded the number of novel venom peptide sequences identified from the species investigated, although most novel conotoxins remain to be pharmacologically characterised. Therefore, efficient high-throughput peptide production systems and/or banks of miniaturized discovery assays are required to overcome this bottleneck and thus enhance cone snail venom bioprospecting and accelerate the identification of novel drug leads.

## 1. Introduction

Millions of years of evolution have generated natural products that can be successfully used as drugs and drug candidates, with >50% of all approved drugs arising from natural products or their derivatives [1]. Although plant-based natural products have been more popular as leads, animal venoms are also regarded as a treasure trove of potential therapeutics [2,3]. Since the success of Captopril, an inhibitor of angiotensin-converting enzyme (ACE) identified from *Bothrops jararaca* snake venom in 1981, drug discovery research using animal venoms has gained momentum [3,4]. Marine cone snails have emerged as one of the most promising sources of new drug leads. This was first shown in 2004 with the FDA approval of Zinconotide, a synthetic form of the ω-conotoxin MVIIA isolated from *Conus magus* used to treat intractable pain [5]. More recently, other conotoxins, including Xen2174 (Mr1A), a norepinephrine transporter antagonist isolated from the *Conus marmoreus* [6], CGX-1007 (Conantokin G), a *N*-methyl-d-aspartate (NMDA) receptor antagonist from *Conus geographus* [7], CGX-1051, a potassium channel blocker from *Conus purpurascens* [8], and ACV1 (Vc1.1) an AChR antagonist identified from *Conus victoriae* [9], have also progressed into the clinic, although only Xen2174 proved efficacious in patients. To enhance the search for successful drug candidates, high-throughput discovery assays are being used in combination with proteomic and transcriptomic approaches and sophisticated structure–activity analyses to accelerate the hit-to-lead process.

Cone snails are marine predators that hunt fish, mollusks, and worms depending on their prey preference, using sophisticated envenomation strategies for prey capture and defense. The ~800 species of cone snails contain a rich variety of small cysteine-rich peptides called conotoxins, together with a lesser number of cysteine-poor conopeptides with diverse pharmacology, that target prey, predator, and/or mammalian receptors with high affinity and specificity [2,10,11]. Cone snail venoms are highly complex with layers of diversity triggered by dietary specialization [12,13] and predatory or defensive stimuli. It has been found that each cone snail species produces a unique venom comprising >1000 venom components [14,15]. Adding to this complexity, variation across individuals of the same species further expands the pool of potentially bioactive peptides available [16,17,18,19,20,21,22].

As drug candidates, peptides possess inherent advantages of higher selectivity and affinity with fewer off-target interactions compared to small molecules, with the promise of better efficacy with potentially lower side effects [23]. Moreover, advanced bio-engineering approaches are now being used to improve the stability and oral bioavailability of peptides [24,25]. In this context, cone snail venom peptides present an ideal source for the mining of potential drug candidates. However, the expected growth in discovery has often been limited by low-throughput, scarcely sensitive discovery methods and material limitations. Typically, abundant peptides from common species such as *C. geographus*, *Conus striatus*, *C. magus*, *Conus catus*, *C. purpurscens*, and *Conus textiles* are most comprehensively studied, while the large pool of minor venom components remain mostly uncharacterized. Tapping into this peptide reserve requires a broader implementation of miniaturised high-throughput multidimensional strategies. This review provides an overview of the impact of these technological advancements on cone snail venom research over the past decade and discusses the challenges and opportunities that remain.

## 2. Venomics: Heralding a New Era of Venom Bioprospecting

Despite cone snail venom peptides being highly regarded as potential drug leads and the diversity and large size of the peptide pool, less than 150 unique peptides have been structurally and pharmacologically characterized over the past 30 years, representing significantly less than 1% of the total conopeptide diversity. To unravel the complex pharmacology of conotoxins and to isolate interesting minor active components using a single venom is challenging as it requires significant quantities of crude venom. Sometimes, tens to hundreds of cone snails are necessary to obtain the venom required to characterize a single molecule of interest [26]. This practice can be a waste of a precious resource and warrants the implementation of miniaturized and accelerated approaches that can better support sustainable bioprospecting.

In recent years, parallel developments in mass spectrometry [27,28,29], next-generation sequencing, [30,31,32] and high-throughput miniaturized target screens [33] have largely benefited venom researchers. For example, adaptation of multi-omics systems approaches to cone snail venom discovery pipelines have enhanced the bio-discovery process and improved our understanding of venom peptide evolution and origin using a small number of animals [15]. By their nature, non-targeted and non-biased transcriptomics and proteomics approaches provide a holistic view of the components that make up cone snail venoms. The integration of these omics approaches to study venoms was termed “venomics” [34]. Since its implementation, cone snail venom research has advanced exponentially with the support of specialized databases (conoserver) [10,11] and automated (conosorter, conodictor) [35,36] and semi-automated analysis pipelines [37] and knowledge bases. Using this integrative technology, full repertoires of venoms were revealed from even rarely studied species, and novel peptide classes and frameworks have been discovered that provide new opportunities for bioprospecting. Perhaps even more interesting, this multi-dimensional approach has helped generate novel hypotheses on venom evolution [14,15,38,39] and cone snail behavior and prey capture strategies [14,20,40,41].

### 2.1. Cone Snail Venom Duct Transcriptomics

In cone snails, the apical secretory cells lining the long, convoluted venom duct [42,43] translates mature mRNA to precursor conopeptides that typically comprise three distinct regions: an N-terminal endoplasmic reticulum (ER) signal sequence, a central pro-peptide region, and the C-terminal mature toxin. Cone snail venom duct transcriptomes reveal the transcripts of all expressed proteins and peptides, including minor peptides and rare variants, providing the template for active venom peptide synthesis during translation [44]. In 2007, the CONCO cone snail venomics project was launched with twenty partnering companies and universities, with the aim of applying this novel approach for the accelerated, cheaper, safer, and more ethical production of innovative biomedical drugs from *Conus consors* and related species [36]. Since then, complete venom transcript repertoires of over a dozen cone snail species have been published using next-generation sequencing platforms such as 454 (Roche, Branford, CT, USA), Illumina (Illumina, San Diego, CA, USA), Ion Torrent™ Personal Genome Machine™ (PGM) (Thermo Fisher, Waltham, MA, USA), Nanopore (Oxford, UK), ABI 3730 Series (Applied Biosystems, Foster City, CA, USA) and PacBio (Pacific Biosciences, Menlo Park, CA, USA) (Table 1).

Initially, the 454 (Roche) pyrosequencing platform was used to obtain the cone snail venom duct transcriptomes [14,15,20,38,40]. However, with the discontinuation of 454 technology in 2013, Illumina is now most widely used to generate venom duct transcriptomes. The transcriptomic data quality is determined by the RNA sequence data read length and the sequencing error rate [45]. Transcriptomes generated through the 454 platform contained longer reads (700 bp single-end) than the Illumina systems (150–300 bp paired-end), though the error rate of illumina is significantly lower than that of the 454 platform (Table 1). The shorter reads generated in Illumina platforms require data assembly [46] using carefully optimized parameters and validation to remove artifacts [44]. This is especially true for cone snail venom duct transcriptome assembly where no reference genome is available as a guide. In contrast, the PacBio system generates longer reads but with a higher error rate that makes this system a poor fit for cone snail transcriptome studies, especially when transciptomic messiness is being investigated [38]. Recently, the complete venom duct transcriptome of *Conus betulinus* [47] ABI 3730 was generated from intermediate length reads (500 bp) with low error rates. As the average length of a conotoxin precursor is ~70 amino acids (~210 nucleotides), platforms offering above 2 × 300 bp nucleotide read coverage with low error rates allow transcriptomes to be faithfully generated.

Irrespective of the platform used, next-generation RNA sequencing generates large data sets that require purpose-built, dedicated bioinformatics tools for efficient data mining. ConoSorter (version 1.1, The University of Queensland, Australia) is one of the most successful standalone tool for rapid and accurate interrogation of thousands of sequences produced by high-throughput sequencing methods. ConoSorter employs Hidden Markov Models for putative toxin identification and annotation. The tool categorizes cDNA or protein sequences into conopeptide superfamilies and classes on the basis of their signal, propeptide, and mature regions. ConoSorter also facilitates the cataloging of the main sequence characteristics (relative sequence frequency, length, number of cysteines, N-terminal hydrophobicity, sequence similarity score) and automatically searches the ConoServer (The University of Queensland, Australia) database of all described conopeptides for known precursor sequences, enabling the rapid identification of known and novel conopeptides. It was reported that, when applied to ConoServer and UniProtKB/Swiss-Prot databases, ConoSorter recognized 100% of known conotoxin superfamilies and classes with a minimum species specificity of 99%, and thus it remains the best publicly available tool for cone snail venom duct transcriptome mining [35].

The growing number of transcriptomic projects have already revealed a wealth of new knowledge about the conopeptides, including the discovery of novel superfamilies and frameworks (Table 2), and have helped uncover novel peptide pharmacology [41,48,49,50,51]. Using this approach, the messy processing at the transcriptomic level was identified as a significant generator of venom peptide diversity [38]. Importantly, messiness is considered a novel mechanism of adaptation in cone snails that contributes to the rapid evolution of venom peptides with new functions.

### 2.2. Proteomics—Exploring the Complexity of Expressed Venom Peptides

The term “proteomics” was first coined in 1995 to define the large-scale characterization of the entire protein profile of a cell line, tissue, or organism [28,56] that underpins the search for a global and integrated view of biology by understanding all proteins and their interactions [57]. Advances in the field of proteomics and the ongoing developments in state-of-the-art instrumentation has greatly benefitted cone snail venom research. Traditional venom peptide identification requires individual peptide isolation by HPLC followed by Edman degradation to identify sequences of individual peptides [15]. Unfortunately, these techniques consume considerable amount of material and are time-consuming. Proteomics provides a versatile approach that supports a comprehensive, multi-pronged analysis of venom peptides, including sequence, quantity, post translational modifications, regionalisation, and stimulus-dependence of venom peptide mobilisation. In cone snail research, proteomics accommodates two main research approaches, i.e., the rapid identification of specific classes of peptides of interest and the systematic big data analysis for comprehensive venomic studies. Large-scale proteomic approaches integrated with matching transcriptomes can help generate novel biological insights by providing a global view of venom complexity beyond the capabilities of classical venom peptide characterization approaches. Understandably, these mass spectrometric methods generate a wealth of data that require comprehensive reference databases and dedicated computational tools to fully exploit their potential.

### 2.3. Mass Spectrometry in Venom Characterisation

Mass spectrometric methods have enabled the characterisation of thousands of different peptides at widely varying concentrations [58] in complex mixtures such as venoms. Liquid chromatography (LC) coupled to mass spectrometry (MS) or tandem mass spectrometry (MS/MS) underpin such proteomic studies [59]. Because of the complex nature of cone snail venoms, it is critical to obtain high-resolution MS data under optimized HPLC conditions to separate and decipher these complex mixtures in a single run. Ultra-high-performance liquid chromatography (uHPLC) and, more recently, microfluidic techniques such as nano-liquid chromatography (Nano-LC) and capillary electro-chromatography (CEC) can deliver remarkable separations using low sample amounts (>2 µg) and flow rates (<1 µL/min) [60]. These techniques provide high selectivity, mass detection sensitivity, separation efficiency, resolution, and rapid analysis and are ideal for the comprehensive proteomics analysis of venoms.

For cone snail proteomic studies, Data-Dependent Acquisition (DDA)-based or Information-Dependent Acquisition (IDA)-based mass spectrometric detection methods are commonly used [15,20,38,48]. The DDA mode selects predetermined *m*/*z* values of interest for secondary MS/MS analysis, providing better ionization of the targeted peptides of interest. IDA serves as an artificial intelligence-based scan mode which provides “on-the-fly” acquisition of MS/MS spectra during a HPLC run to obtain specific, selective, and information rich MS/MS from precursor ions [55]. Not surprisingly, these methods have greatly facilitated the generation of MS/MS lists of peptides from crude cone snail venoms for database searches and sequence validation. Time-of-flight (TOF) mass spectrometry, especially quadrupole–time-of-flight (Q-TOF) MS, provides fast acquisition speeds, superior sensitivity, high resolution, and excellent mass accuracy [43], characteristics that are all required for venomics studies. For example, by using these state-of-the-art techniques it was found that each crude venom comprises >1000 peptides, while traditional peptide detection methods typically found only ~100 peptides [15,20,38,48].

Comprehensive MS/MS libraries of venom peptide fragments facilitate the integration with venom duct transcriptomic data, allowing the relatively straightforward assignment of full sequences for many of the peptides present in the proteome, in a bottom-up proteomics approach [58]. Typically, cone snail venoms are digested into peptide fragments by a sequence-specific enzyme such as trypsin or Glu C, and MS/MS spectra (IDA- or DDA-assisted) are generated that contain sufficient sequence information to identify and quantify the parent peptides [15,20,38]. Collision-induced dissociation or higher-energy collisional dissociation [61] methods are often used for ion fragmentation. However, alternative methods such as electron transfer dissociation [62] are becoming more widely available and promise improved fragmentation of larger and modified peptides. These novel methods can be readily incorporated into the cone snail venom peptide identification pipeline for high-throughput peptide identification and validation.

### 2.4. Omics Data Integration

The use of omics data to interpret complex proteinaceous compositions became possible with the introduction of the comprehensive databases and efficient database searching algorithms [57]. These databases and search tools enable the integration of transcriptomic data into the proteomic data and into a powerful pipeline for rapid peptide identification and quantification. However, there are few studies that reveal close to the full set of toxins produced in the venom gland of even a single species. To date, the deep-venomics study of *C. marmoreus* still remains the most comprehensive, with 60% of the transcriptome (105 precursors) explained by 1385 peptide fragments sequenced by MS/MS [15,35]. Despite the challenges in finding all major transcriptomic sequences in the proteome, venomics remains the fastest and most efficient approach to obtain broad-scale sequence data, even for highly divergent peptides of minor gene superfamilies [15,20,35,38,40,49]. The major bottleneck in venomic data analysis and interpretation is the lack of dedicated reference databases and robust data analysis tools that can integrate all aspects of conotoxin diversity and complexity. Currently, UniProt, Conoserver, and venom transcriptome sequence libraries serve as reference databases for proteomic search, with software commonly used to validate transcriptomic sequences against tandem mass spectrometric data listed in Table 3. As the field of proteomics continues to advance, novel high-resolution mass spectrometry in combination with new dedicated venom databases and optimized search engines are expected to improve the sequence recovery rates. User-friendliness, reliable post-translational modification (PTM) prediction and scoring, and improved data visualisation tools are expected to facilitate venom peptide proteome and transcriptome integration and help unravel the underlying biology driving their remarkable diversity.

## 3. Complex Venom Processing Revealed through Multi-Omics Studies

Cone snail venom peptides frequently carry a diverse range of PTMs, with up to 75% of amino acids post-translationally modified in a single conotoxin. This heavy and unpredictable incorporation of PTMs often confounds the automated integration of transcriptomic and proteomic data. Current search engines, such as ProteinPilot™ (Sciex, Washington, DC, USA) and the Conomass tool of Conoserver, enable the detection of common conotoxin PTMs including disulfide bonds, C-terminal amidation, pyroglutamylation, glutamate carboxylation, proline hydroxylation, valine hydroxylation, tryptophan bromination, and tyrosine sulfation. However, the identification of complex PTMs like glycosylations is not facilitated by these methods, and their presence usually requires de novo sequencing support. The development of algorithms to facilitate automated PTM identification and verification is needed to facilitate integration, especially for the analysis of big datasets.

Despite these challenges, completed cone snail venomic studies have disclosed an unprecedented level of peptide processing beyond PTMs, including variable peptide processing and transcriptomic messiness. In the first *C. marmoreus* venomic study, 7798 unique masses were identified in the proteome, while the venom duct transcriptome revealed only 105 conopeptide coding sequences. Through deep proteomic interrogation, Sebastien et al. uncovered a new mechanism of variable peptide processing (VPP) that contributes to the remarkable diversity of conopeptides [15]. This study highlighted an extensive and highly variable processing of the *N*- and *C*-termini of the mature conopeptides that dramatically increased venom peptide diversity. This variable peptide processing, together with PTMs, explains how a limited set of gene transcripts can generate thousands of conopeptides in the venom of a single species of cone snail.

In addition to the variable processing of peptides after translation, a layer of transcriptomic variability was found to arise from low-level transcriptomic variability. Jin et al. found that a surprisingly large number of validated cone peptide gene sequences expressed at low levels contributed to transcriptomic “messiness” and venom hyper-variability [38]. Supporting this variability, Lu et al. found that conotoxin-encoding transcripts can be diversified via hypermutations, fragment insertion/deletions, and mutation-induced premature terminations that allow a single mRNA species to generate multiple toxin products [63]. Thus, both genetic and post-translational messy processing of the venoms contribute significantly to an expansion of conopeptide complexity. All these mechanisms of peptide processing provide a nascent pool of accumulated chemical diversity that can facilitate the rapid evolution of venom peptides with new functions and likely underlies the rapid adaptive radiation found in cone snails.

### Proteomic Data Visualization Reveals a Remarkable Venom Variability

**Figure 1:**
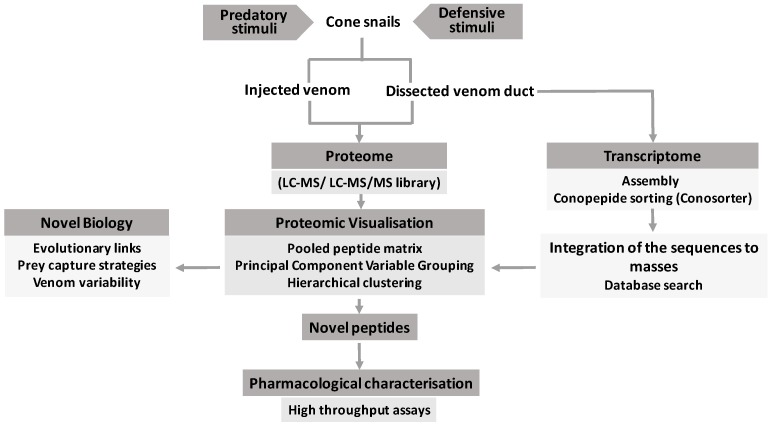
A rational venomic approach integrating proteomic data visualization approaches to accelerate the comparisons of complex venoms and the rapid identification of likely functionally relevant novel peptides.

Extensive variation in cone snail venom composition is well documented to occur at nearly all biological scales from venom duct regionalisation, to individual variability and species differences. Understanding this variation in venom composition not only would reveal the possible forces that shape venom evolution, but also has implications for venom peptide pharmacology and an understanding of protein structure–function relationships [13]. To better understand this variability, extensive proteomic profiling has been carried out in the past decade [16,17,18,19,20,21]. The possibility of mass spectra and tandem mass spectra to be readily incorporated into the bioinformatics tools has opened up an exciting opportunity to rapidly profile and visualize this variability to reveal any underlying patterns of peptide expression. Triple-TOF-MS and MS/MS peptide quantification algorithms (Table 3) are increasingly popular for the rapid analysis of complex samples such as venoms. These algorithms, along with clustering techniques such as principal component analysis (PCA) [64,65], hierarchical clustering, molecular networking, and heat-map visualization, provide invaluable tools to gain new insights into venom peptide expression patterns. While it is still early days for data visualization of venoms, this approach has been successfully applied to better understand other complex proteomes as part of a systems biology approach [66,67,68]. This approach can enrich the integrated venomics approach outlined in Figure 1 to enhance the identification of promising novel peptides that can then be purified or synthesized to discover their functionality.

**Figure 2:**
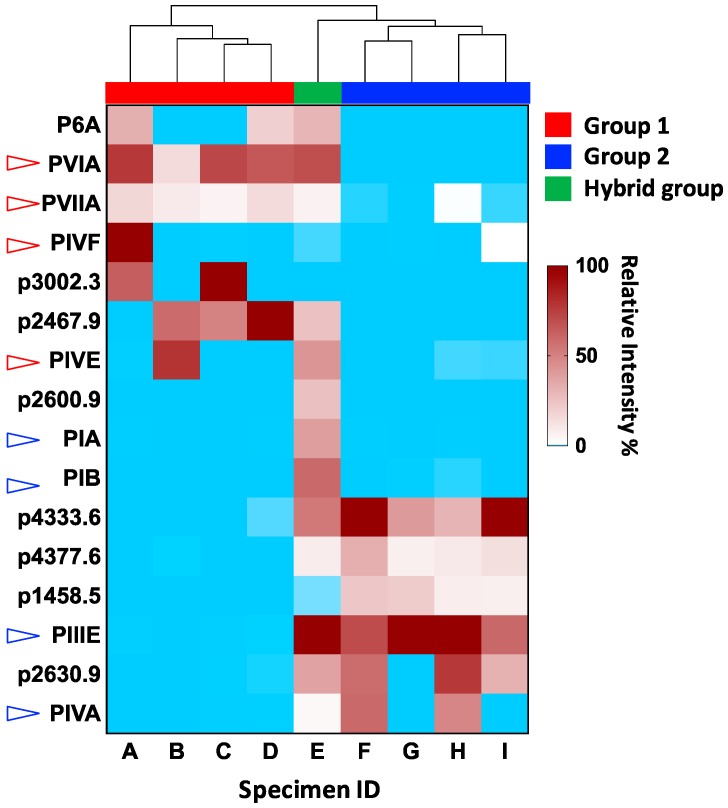
Proteomic visualisation of the known peptides and most abundant novel peptides of the injected predatory venom collected from nine individual specimens of *C. purpurascens*. The heatmap matrix shows peptides that co-cluster with each of the three groups, revealing remarkable venom variability across these specimens. The red, open arrows indicate known excitatory peptides, and the blue, open arrows indicate known neuromuscular blockers. The relative intensity of each peptide is shown in a gradient of brown, with the absence of peptide indicated by the blue color. This figure is adapted from Himaya et al. 2018.

The first attempt at automating cone snail venom proteomic visualization examined the individual predation-induced venoms from nine *C. purpurascens* [22] using MarkerView™ (Sciex, Washington, DC, USA) to compare and cluster. The downstream analysis with PCA and hierarchical clustering revealed that two major prey-capture cabals (defined sets of synergistic venom peptides) found in this species were typically employed independently and not simultaneously as previously suggested. Interestingly, this clustering approach also revealed separate sets of novel venom peptides associated with either the excitatory or the inhibitory cabals that may help unravel their biology and evolutionary links (Figure 2). While this approach requires better linking to transcriptomic data, it provides a rational and accelerated approach to interrogate the increasing amounts of multi-omics data provided by next-generation proteomics and transcriptomics.

## 4. Concluding Remarks

An important goal of cone snail venomic research is to accelerate the discovery of structurally diverse and pharmacologically valuable venom peptides and to help unravel their evolutionary trajectories. Already, venomics approaches have identify and understood key evolutionary innovations of: (i) expanded venom peptide diversity through variable peptide processing and transcriptomic messiness, (ii) regionalization of the venom duct for distinct predatory and defensive functions, and (iii) piscivory and its likely origins from repurposed ancestral worm hunting defensive venoms. However, despite the expansion of sequence information, the rapid and selective pharmacological characterisation of conotoxins and the identification of complex PTMs remain a challenge. To take advantage of the thousands of validated transcriptomic sequences available, a streamlined data-mining, production, and characterization pipeline is required that integrates high-throughput structural and pharmacological characterization with accelerated peptide production systems. Recently, a promising high-throughput recombinant expression of di-sulfide-reticulated venom peptides that can generate large libraries as an alternative to chemical synthesis was reported [69] that may start to fill this growing gap in the pipeline.

## Figures and Tables

**Table 1:** Performance of next-generation sequencing platforms.

Developer	System	Read Length (bp)	Error Rate (%)
Illumina	Illumina Hi-Seq2000	2 × 150 bp	0.1
	Illumina Hi-Seq3000/4000	2 × 150 bp	0.1
	Illumina Mi-Seq (Version 3)	2 × 300 bp	0.1
Thermo Fisher Scientific	Ion Torrent PGM 318	400 bp	1
Applied Biosystems	ABI 3730/ABI 3730xl	500 bp	0.4
Pacific Biosciences	PacBio RS II	10–20 kb	10–15
	PacBio Sequel	>20 kb	10–15
Oxford	Nanopore PromethION	900 kb	4
Roche	454 (discontinued in 2013)	700 bp	1

**Table 2:** New superfamilies discovered by transcriptomics.

Species	New Superfamily	Cysteine Framework	Sequencing	Reference
*Conus arenatus* *Conus coronatus* *Conus rattus*	MRFYM-	VI/VII	Illumina HiSeq 2000	[13]
*Conus arenatus* *Conus imperialis* *Conus lividus* *Conus quercinus* *Conus sponsalis* *Conus virgo*	MKISL-	VI/VII	Illumina HiSeq 2000	[13]
*Conus marmoreus*	N, B, H, E, F, H2, I4, M2, N2, O4, Q, R, U, W, X, Y2, Y3, Z	XV, VIIII, VI/VII, N, N, III, C1, C7 *, N, VI/VII, N, N, VI/VII, N, C2, C4 *, C2, C2	Roche 454	[15]
*Conus catus*	Cat-NSF1	VI/VII	Roche 454	[20]
*Conus miles*	SF-mi 1–8	XIII, C8-novel, VI/VII, N, N, XV, NA, XIII	Roche 454	[38]
*Conus vexillum*	NSVx1-4	XIV, XXIV, VI/VII, XXVIII (novel)	Roche 454	[40]
*Conus betulinus*	NSF-bt01–09	IX, XV, VI/VII, XIV, VI/VII, IX, VIII, VI/VII, IX	ABI 3730	[47]
*Conus episcopatus*	SF-Epi 1–16	variable, III ^a^, V, C7 *, V, V, V, XI, C7 *, N, C5 *, NA, XVIII, VI/VII, IV, C5 *	Illumina MiSeq	[48]
*Conus tribblei and Conus lenavati*	SF-01-04	C12 *, C12 *, IX, XIII,	Illumina HiSeq 2000	[49]
*Conus pullicarius*	J2	XIV	Roche GS-FLX	[52]
*Conus gloriamaris*	MKAVA-, MSRLF-, MMLFM-, MLSML-	XXII, N, VIII, C12 *	Illumina HiSeq 2000	[53]
*Conus lenavati*	SF-05-06	XIV, C12 *	Illumina HiSeq 2000	[54]
*Conus andremenezi*	Put.MGGRF, Put.MKAVA	N, C8 *	Illumina HiSeq 2000	[55]
*Conus andremenezi Conus praecellens*	Put.MSGLR, Put.MUSGK	VI/VII, C6 *	Illumina HiSeq 2000	[55]

N, no cysteines were detected in the mature sequence; * no framework name has been defined, possibly a novel cysteine framework; ^a^ probable predicted framework.

**Table 3:** Commonly used search algorithms and proteomic analysis tools. PTM: post-translational modification.

Tool	Function
**Database search algorithms**	
PEAKS DB	Database search engine, run in parallel with de novo sequencing, to automatically validate the search results
ProteinPilot Software	Enables peptide identification while considering PTMs, non-tryptic cleavages, and amino acid substitutions. Supports quantification using iTRAQ, mTRAQ, and SILAC
Mascot	Peptide mass fingerprinting and MS/MS database searching
Protein Prospector	Proteomic analysis tools including “Batch-Tag” for instrument- and fragmentation mode-optimised analysis
MassMatrix	Search algorithm for tandem MS data that ranks peptide and protein matches
Byonic	MS/MS data search for fragment identifications to produce protein scores and identification probabilities
**MS/MS peptide quantification and visualisation software**	
MarkerView Software	Statistical analysis and visualisation of quantitative mass spec data sets from proteomic profiling applications
MultiQuant Software	Quantitation and targeted visualisation of TripleTOF or QTRAP data, including MRM and SWATH acquisition
MaxQuant	Quantitative proteomics tool for analysis of label-free and SILAC-based proteomics data

## Abbreviations

PTM	Post-translational modification
ACE	Angiotensin-converting enzyme
LC/MS	Liquid chromatography/mass spectrometry
u-HPLC	Ultra-high-performance liquid chromatography
Q-TOF-MS	Quadrupole-time-of-flight mass spectrometry
DDA	Data-dependent acquisition
IDA	Information-Dependent acquisition
VPP	Variable peptide processing
PCA	Principal component analysis
